# High Surface Area Mesoporous Silica Nanoparticles with Tunable Size in the Sub-Micrometer Regime: Insights on the Size and Porosity Control Mechanisms

**DOI:** 10.3390/molecules26144247

**Published:** 2021-07-13

**Authors:** Federica Rizzi, Rachele Castaldo, Tiziana Latronico, Pierluigi Lasala, Gennaro Gentile, Marino Lavorgna, Marinella Striccoli, Angela Agostiano, Roberto Comparelli, Nicoletta Depalo, Maria Lucia Curri, Elisabetta Fanizza

**Affiliations:** 1 Department of Chemistry, University of Bari, via Orabona 4, 70126 Bari, Italy; federica.rizzi@uniba.it (F.R.); pierluigi.lasala95@gmail.com (P.L.); angela.agostiano@uniba.it (A.A.); 2 Institute for Physical Processes, Italian National Research Council, c/o Department of Chemistry, University of Bari, via Orabona 4, 70126 Bari, Italy; m.striccoli@ba.ipcf.cnr.it (M.S.); r.comparelli@ba.ipcf.cnr.it (R.C.); n.depalo@ba.ipcf.cnr.it (N.D.); 3 Institute for Polymers, Composites and Biomaterials, Italian National Research Council, via Campi Flegrei 34, Pozzuoli, 80078 Naples, Italy; rachele.castaldo@ipcb.cnr.it (R.C.); gennaro.gentile@ipcb.cnr.it (G.G.); 4 Department of Bioscience, Biotechnology and Biopharmaceutics, University of Bari, via Orabona 4, 70126 Bari, Italy; tiziana.latronico@uniba.it; 5 Institute for Polymers, Composites and Biomaterials, Italian National Research Council, Piazzale E. Fermi 1, Portici, 80055 Naples, Italy; marino.lavorgna@cnr.it

**Keywords:** mesoporous silica nanoparticles, high specific surface area, colloidal synthesis

## Abstract

Mesoporous silica nanostructures (MSNs) attract high interest due to their unique and tunable physical chemical features, including high specific surface area and large pore volume, that hold a great potential in a variety of fields, i.e., adsorption, catalysis, and biomedicine. An essential feature for biomedical application of MSNs is limiting MSN size in the sub-micrometer regime to control uptake and cell viability. However, careful size tuning in such a regime remains still challenging. We aim to tackling this issue by developing two synthetic procedures for MSN size modulation, performed in homogenous aqueous/ethanol solution or two-phase aqueous/ethyl acetate system. Both approaches make use of tetraethyl orthosilicate as precursor, in the presence of cetyltrimethylammonium bromide, as structure-directing agent, and NaOH, as base-catalyst. NaOH catalyzed syntheses usually require high temperature (>80 °C) and large reaction medium volume to trigger MSN formation and limit aggregation. Here, a successful modulation of MSNs size from 40 up to 150 nm is demonstrated to be achieved by purposely balancing synthesis conditions, being able, in addition, to keep reaction temperature not higher than 50 °C (30 °C and 50 °C, respectively) and reaction mixture volume low. Through a comprehensive and in-depth systematic morphological and structural investigation, the mechanism and kinetics that sustain the control of MSNs size in such low dimensional regime are defined, highlighting that modulation of size and pores of the structures are mainly mediated by base concentration, reaction time and temperature and ageing, for the homogenous phase approach, and by temperature for the two-phase synthesis. Finally, an in vitro study is performed on bEnd.3 cells to investigate on the cytotoxicity of the MNSs.

## 1. Introduction

Silica-based mesoporous nanoparticles (MSNs) have attracted increasing attention as nanocontainers [1,2,3] thanks to their high specific surface area, easy and versatile surface chemistry modification [4], narrow pore size distribution, tunable characteristics of pore network, with pore size ranging from 2 to 50 nm, and excellent biocompatibility with minimal non-specific or adverse effects [5,6]. These features make these structures potential candidates for delivery of active payloads useful in several fields of application [7] including catalysis [8], separation [9], energy storage [10], corrosion protection [11] and in biomedicine [12], as drug reservoir and bioimaging platforms. 

Most of the synthetic approaches used to prepare MSNs derive from the pioneering work of Stöber [13], based on a sol-gel process of an alkoxysilane in water-alcohol solution, at ambient conditions in the presence of ammonia, as base catalyst, and modified via addition of a pore-structuring agents. In general, alkaline, as well as acidic, conditions [1] have been exploited to catalyze hydrolysis and condensation of a silica precursor that self-assembles with the organic structure directing agents (SDA), i.e., surfactants, co-surfactants and swelling agents. Therefore, formation of the MSNs results from a judicious combination of templating methods and inorganic sol-gel procedures. Most common methodologies [14] are based on quaternary alkylammonium surfactants (i.e., cetyltrimethylammonium bromide, CTAB) as SDA, and are performed under strongly basic conditions, in the presence of tetraethyl orthosilicate (TEOS) as silica source, resulting in porous structures with pores size of nearly 3 nm. In addition, MSNs with controlled morphology, surface area, pore size, and pore channels orientation have been obtained by properly tuning the synthesis conditions, like surfactant composition, concentration and counter ions, and their self-assembly [15]. It has been also found that the nature of silica precursors, solvents or co-solvents, catalysts, and, finally, pH of the reaction medium also affect the MSN structure. Spherical worm-like MSNs with mesopores or hierarchical porous structures, including hollow [16,17,18], yolk-shell, stellate nanoparticles (NPs) [19] have been synthesized by suitably tuning SDA, precursors nature, concentration, reaction condition such as duration and temperature of hydrolysis and condensation or application of post-synthetic treatments. 

A large body of recent literature [16,20,21,22,23] on the MSNs synthesis mainly covers aspects concerning the capability of tailoring, by modulating solution phase conditions, the pore size, shape and connectivity towards enhanced loading efficiency of active payloads. However, the control of particle size, specifically below 200 nm, remains challenging, due to the easy tendency of MSNs to aggregate. Indeed, MSN size regime between 50–200 nm, together with monodispersity, controlled pore size, shape and surface chemistry, represent essential features for biomedical application of MSNs as drugs and contrast agent vehicles [24,25]. Several studies have showed that efficiency of the cellular uptake and possibility to penetrate through the membranes/tissue are strongly dependent on particle size. Particle size has a relevant impact on toxicity and biodistribution of the material, and on delivery rate to targeted cells. MSNs with up to sub-micrometer size have been reported to provide enhanced cellular uptake by endocytosis, that is an essential feature to achieve high efficiency in biological applications, avoiding fast exocytosis, typically occurring for very small MSNs (<50 nm) [12]. In fact, the actual size threshold favoring cellular uptake is still debated, as in some cases superior uptake performance has been evaluated for smaller MSNs rather than larger particles, while, in other cases, larger particles, around 100–150 nm, have been found to be more effectively taken-up than the smaller ones [6,12]. In addition, surface properties such as functional groups and surface potential (hence, surface charge) can influence the biocompatibility of MSNs. In this regard, it has been reported the critical role played by the (-SiOH) groups at the surface layer of MSNs. These surface functional groups can interact with biological molecules, inducing generation of reactive oxygen species (ROS) [5,6,26]. Cells viability and cytotoxicity of MSNs have thus shown size- and surface properties dependence [12]. 

Typically, control on MSN size within the sub-micrometer regime, is achieved by replacing ammonia, which is conventionally used as basic catalyst in the Stöber process, with organic amines. They are found to effectively contain MSN size below 200 nm, since they act not only as catalyst but also as particles blocking agents, thanks to their interaction with silica surface. Such organic amines are able to catalyze the sol-gel reaction under mild basic condition, and, at the same time, prevent particle aggregation [15]. In particular, the use of triethanolamine (TEA) for synthesizing MSNs with diameter ranging between 20 and 150 nm has been widely reported [27]. It sets reaction mixture pH at 9–10, required for the hydrolysis of alkoxides precursors and growth of porous structures, meanwhile its chelating behavior limits the MSNs growth [27]. However, the use of such mild base usually requires high reaction temperature. 

When NaOH is used to catalyze the synthesis of MSN, typically a high reaction temperature (80–100 °C) and a large volume of aqueous base (200–1000 mL) are required [28,29,30,31,32,33,34,35,36,37,38,39,40,41,42,43,44,45,46,47,48] to contain MSN size in sub-micrometer regime [49]. In fact, such a temperature range, required to trigger nucleation and MSN formation, results in the formation of large, micrometer-sized, silica particles aggregates, and the use of large volume of aqueous NaOH limits particle aggregation and uncontrolled formation of larger MSNs thanks to dilution. Therefore, the proper combination of high temperature and a large volume of aqueous base speed up the silica precursor hydrolysis, avoiding the nucleation while controlling the growth.

Herein, we have addressed the issue arising from the use of NaOH as catalyst for the formation of sub-micrometer (<150 nm) sized MSNs, specifically aiming to develop a synthesis maintaining a low reaction volume and moderate temperature, with a view to achieving higher sustainability. In particular, two synthetic approaches have been studied which rely, respectively, (i) on a homogeneous aqueous/ethanol-containing reaction mixture, and (ii) a two-phase aqueous/ethyl acetate-containing system, both based on CTAB as SDA, TEOS as silica source and NaOH as catalyst. The reaction temperature has been kept no higher than 50 °C (30 °C and 50 °C, respectively). The two strategies have been compared, morphologically and chemically, by investigating the resulting particles as a function of catalyst concentration, reaction temperature and time, to elucidate the mechanism that regulates the control of MSN size and size distribution. The obtained results demonstrate that an effective tuning of the MSN size in the range of 40–150 nm can be attained as a function of NaOH concentration, reaction time and temperature and ageing when the homogenous solution approach is followed, while when the two-phase system is used it is the reaction temperature that mainly controls the size of MSNs.

## 2. Results and Discussion

### 2.1. Synthesis of MSNs: Homogenous Solution Approach

**Figure 1:**
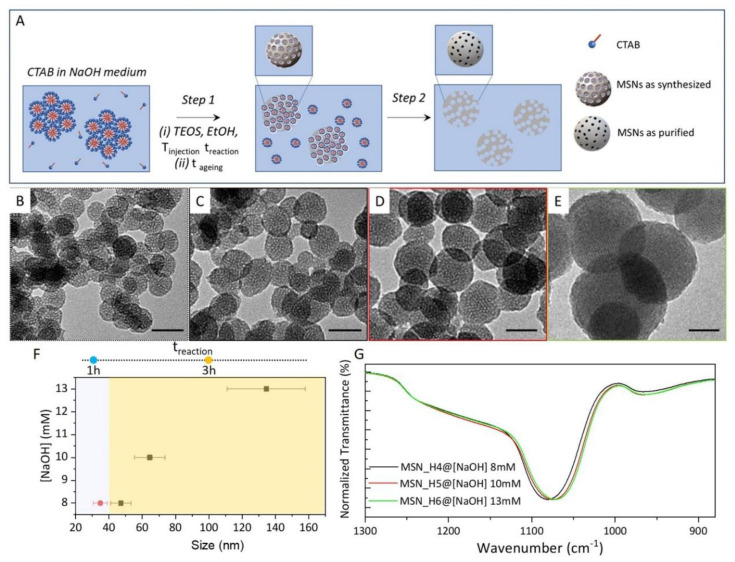
(**A**) Sketch of the synthetic MSNs in homogeneous solution. Step 1: (i) EtOH and TEOS are added to CTAB micellar aqueous solution at alkaline pH by NaOH at injection temperature, T_injection_, the reaction mixture is let stirring for different reaction time (t_reaction_) then (ii) the temperature is decreased at room temperature (t_ageing_) to allow MSNs growth. Step 2: surfactant template removal; (**B**–**E**) TEM micrographs (scale bar 50 nm) of MSNs (see also Table 1) MSN_H3 (**B**), MSN_H4 (**C**), MSN_H5 (**D**) and MSN_H6 (**E**); (**F**) Scatter plot of MSN average size, including size distribution as function of NaOH concentration and t_reaction_; (**G**) FTIR spectra recorded in ATR mode in the 1300–880 cm^−1^ range of MSNs samples (panel (**F**)).

Figure 1A shows the scheme of the MSNs’ preparation in the homogenous aqueous/ethanol solution, by using CTAB micelles as SDA, NaOH as catalyst and TEOS as alkoxysilane precursor. A basic, aqueous, 5 mM CTAB solution, which is above the critical micellar concentration, and characterized by hexagonal structures, has been heated up at T_injection_ and ethanol and TEOS have been added, under stirring, resulting in a homogenous colloidal solution (Figure 1A, Step 1(i)). According to the commonly reported procedures (see reactions (1) and (2) below), the basic pH provided by NaOH promotes the hydrolysis of TEOS, bursting the nucleation of the MSNs and supporting the further condensation of Si-O-Si networks, while the presence of CTAB micelles, interacting with the silica precursor, templates mesopores formation. The obtained MSNs have been aged at room temperature (Figure 1A, Step 1(ii)) for a defined time (t_ageing_), under stirring, prior their separation from the reaction mixture by centrifugation. A solvent extraction procedure, based on the use of ethanol added with a small amount of HCl (see the Materials and Methods section) allows to remove the pore-forming CTAB micelle and to finally recover purified MSNs (Figure 1A, Step 2).

Si(OEt)_4(aq)_ + 4H_2_O_(l)_→Si(OH)_4(aq)_ + 4EtOH_(l)_ (1)

(HO)_3_Si-OH_(aq)_ + HO-Si(OH)_3(aq)_→(OH)_3_Si-O-Si(OH)_3(aq)_ + H_2_O_(l)_ (2)

**Figure 2:**
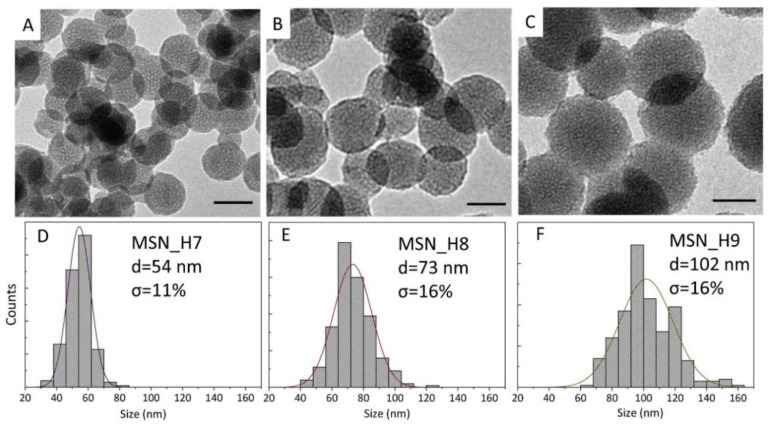
TEM micrographs ((**A**–**C**), scale bar 50 nm) and the corresponding size distribution statistical analysis (**D**–**F**) of MSN_H7 (**A**,**D**), MSN_H8 (**B**,**E**) and MSN_H9 (**D**,**F**).

The prepared samples have been characterized by TEM (Figure 1B–E and Figure 2A–C, Figures S1 and S3 in the Supplementary Materials), also performing a size statistical analysis (Figure 2D–F and Figure S2 in the Supplementary Materials). The synthetic conditions used for the preparation of each sample, and the average size and size distribution are summarized in Table 1. 

In particular, the synthesis of MSNs has been performed by varying (i) NaOH concentration, namely 5, 8, 10 and 13 mM, (ii) injection temperature (T_injection_), 50 °C and 30 °C, (iii) keeping the reaction medium at this temperature condition for 1 hour or 3 h (t_reaction_), and (iv) subsequently letting it age (t_ageing_) for 4 h or 24 h at room temperature. Remarkably, here, a maximum T_injection_ of 50 °C and only small H_2_O volume (≤ 50 mL) have been used, both much lower than those conventionally employed for the synthesis of MSNs in aqueous medium. Such conditions result advantageous, in terms of energy consumption and sustainability, from the perspective of an eventual technological upscaling of the synthesis.

A first set of experiments has been carried out at T_injection_ = 50 °C, in a H_2_O volume of 50 mL and with a t_ageing_ of 24 hours, varying NaOH concentration and t_reaction_. As shown by the TEM micrographs reported in Figure 1B–E, the MSNs prepared using 8mM (MSN_H3 and MSN_H4), 10 mM (MSN_H5) and 13mM (MSN_H6) NaOH, respectively, present a spherical morphology, sub-micrometer size with porous structure and a rough surface. Conversely, using 5 mM NaOH (MSN_H1 and MSN_H2), irrespective of the reaction mixture composition, an interconnected matrix, rather than isolated particles, has been obtained (Figure S1 in the Supplementary Materials), probably due to a pH not sufficiently basic to catalyze the formation of MSNs. 

MSNs prepared using 8 mM NaOH exhibit an average diameter of 35 nm (σ% = 11%, MSN_H3 sample, Figure 1B, Figure S2A in the Supplementary Materials) after 1h of reaction, that increases up to 47 nm (σ% = 13%, MSN_H4 sample, Figure 1C, Figure S2B in the Supplementary Materials) after 3 h of reaction, therefore a shorter t_reaction_ affords smaller MSNs. Extending the t_reaction_ beyond 3 h only a negligible increase in the average size has been observed. However, larger MSNs can be obtained by further increasing NaOH concentration: using 10 mM NaOH (Figure 1D, MSN_H5 sample, Figure S2C in the Supplementary Materials) MSNs with an average diameter of 64 nm (σ% = 15%) are achieved that get even larger, 135 nm diameter (σ% = 17%) using 13 mM NaOH (Figure 1E, sample MSN_H6 sample, Figure S2D). As summarized in Table 1 and displayed in Figure 1F by the reported error bars, a broadening of size distribution is observed, with σ% going from 13% to 17%, when the NaOH concentration increases from 8 mM up to 13 mM. In addition, when the MSNs are aged for 4 h (Figure 2A,D), as in the case of MSN_H7, particles of 54 nm diameter (σ% = 11) are obtained, smaller than MSN_H6 (average size 135 nm and σ% = 17%), aged for 24 h. This result indicates that ageing step significantly contribute to the MSNs growth. 

Description of silica particles in terms of structures and porosity has been widely based on the analysis of IR spectra [50,51,52,53,54,55]. IR studies are particularly valuable because of the high polarity of silicon-oxygen bonds. For silica particles of small size and with high specific surface area the O-H and Si-O stretch IR absorptions of silanol (SiOH) groups at 3750–3650 cm^−1^ and 960 cm^−1^, respectively, Si-O stretch absorptions of siloxide (SiO-) groups nearly at 1000 cm^−1^ and the antisymmetric stretch absorptions of surficial siloxane bridge groups at 880 cm^−1^ are of valuable importance. The intense absorption bands in the 1300–1000 cm^−1^ region, usually is characterized by a contribution ascribed to the transverse optical (TO) modes of the lattice, and a shoulder at higher wavenumber characteristic of the longitudinal optical (LO) mode. Their relative intensity and position have been used to obtain information concerning strains on the Si–O–Si bonds. Thus, FTIR investigation in the 1300–880 cm^−1^ range of MSNs can provide relevant insights on their structure [50,55]. The FTIR spectra in ATR mode of MSN_H4 (Figure 1G black line), MSN_H5 (Figure 1G red line) and MSN_H6 (Figure 1G green line), prepared at increasing NaOH concentration, highlight a progressive red-shift of the transverse-optical mode of Si-O-Si asymmetric stretching vibration band, that moves from 1081 cm^−1^ for MSN_H4 (prepared using 8mM NaOH) to 1071 cm^−1^ for MSN_H6 (prepared using 13 mM NaOH). Indeed, according to the vibration equation ($v\propto k^{\frac{1}{2}}$, where *v* is the wavenumber and *k* is the force constant between linked atoms), the red shift of the Si-O-Si peaks reveals a decreasing binding energy and increasing distance of the Si-O bond in the network. Therefore, the red-shift of the Si-O-Si band suggests that a more open structure is formed, with lower compressive stress, thus vibrations can occur more freely in the MSNs prepared using higher NaOH concentration. Such a result indicates a lower cross-linking degree for MSN_H6 sample compared to MSN_H4. Concomitantly, the relative intensity of the Si-OH stretching vibration at 965 cm^−1^ is higher for the MSN_H6 than in the other samples, thus suggesting the presence of a larger amount of silanol groups. 

**Figure 3:**
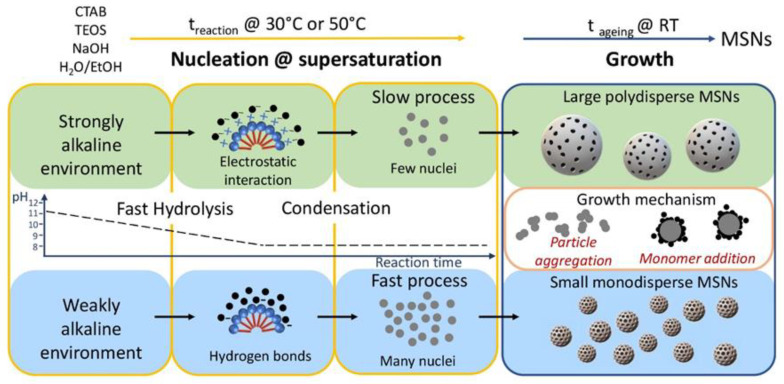
Scheme of the pH-dependent nucleation and growth of the MSNs, prepared in homogenous solution. pH value controls the kinetic of hydrolysis and condensation during the nucleation at high reaction temperature (t_reaction_), and the growth step at room temperature.

A reaction scheme has been depicted and reported in Figure 3 to account the size control of MSNs as a function of the alkaline environment condition. Indeed, pH influences the hydrolysis and condensation reaction [56]. Hydrolysis rate reaches its maximum at basic pH, while condensation rate at pH close to neutrality, decreasing at higher and lower pH [56]. Similarly, also MSN growth is affected by pH, by defining the particle surface potential. Therefore, by measuring the medium pH along the reaction it is possible to monitor the different steps of the synthesis, obtaining insights into the mechanism, and thus enabling a careful control of the MSNs’ size. 

In addition, in the CTAB-mediated MSN synthesis, following the hydrolysis of TEOS catalysed by NaOH, a complex is formed between hydrolysed TEOS and CTAB micelles, which is cooperatively stabilized by hydrogen bonds and weak electrostatic interactions at weakly basic pH, while only electrostatic interactions are responsible of its stability under strongly alkaline conditions. Such complexes represent the monomer feeding the MSNs synthesis.

In our experiments, CTAB micelles form in a reaction mixture having pH of 11.8, 12.0 and 12.1 by 8, 10 and 13 mM NaOH, respectively, that decreases down to 8.2, 8.6 and 9.0, respectively, upon addition of TEOS and ethanol. Such a decrease in pH can be ascribed to consumption of hydroxyl ions due to the hydrolysis of TEOS. 

When, at this stage, the pH is close to neutrality, as for the synthesis of MSN_H3 and MSN_H4 prepared by using 8 mM NaOH, it is reasonable to assume that the initial hydrolysis step lasts a short time and is soon followed by condensation of the siloxane bond [56]. For this set of samples, due to the starting pH condition, the hydrolysis, which supplies the fresh monomer, becomes soon very slow while condensation of silicate species proceeds fast, forming the Si-O-Si network and, subsequently, the primary particles. Since the condensation step takes place at the early stage of the reaction, that is at high temperature, a high degree of condensation is expected, as also confirmed by the FT-IR characterization. 

Conversely, when 13 mM NaOH is used, the pH measured for the reaction mixture results still high enough (pH = 9.0) after TEOS and ethanol addition, thus turning in a fast hydrolysis and a slow condensation, both taking place during the t_reaction_. 

Indeed, as highlighted in the scheme in Figure 3, the nucleation, which forms the primary particles, lasts until supersaturation conditions are fulfilled. According to the classical nucleation theory of LaMer, when the concentration of hydrolysed TEOS drops below the critical supersaturation, nucleation stops, and particles start to grow until primary particles first, and then final, stable, colloidal particles are formed [57]. Two mechanisms have been generally suggested for the growth of silica nanoparticles: the former based on monomer deposition on the already formed particles and the latter on particle self-assembly/aggregation. The latter usually occurs at pH values close to neutrality, since conversely basic pH generates a negative charge density on the silica surface, hindering particle aggregation due to surface electrostatic repulsion.

Based on these considerations, for MSN_H3 and MSN_H4, the slow monomer supply, due to slow hydrolysis, is expected to quickly establish a monomer concentration below supersaturation, so that MSNs would begin to grow. After 3 h reaction time, the pH is found to increase (pH = 9.5), remaining then constant until the end of the reaction. Such a pH condition sustains a growth based on monomer deposition, rather than primary particles assembly/aggregation. However, the slow availability of monomer keeps small the average size of MSN_H3 and MSN_H4, with size distribution that slightly broadens as t_reaction_ increases, probably due to monomer consumption and establishing of Ostwald ripening regime. 

For MSN_H6, the pH of the reaction medium after 3 h at 50 °C is 8.2, close to neutrality, thus suggesting the consumption of hydroxyl radicals and thus confirming the occurrence of hydrolysis throughout the reaction time. The pH neutrality, during the ageing step, indicates a growth mechanism mediated by primary particle self-assembly or aggregation, until a colloidally stable size is reached. Indeed, the aggregation-mediated growth is also supported by the increase in the ionic strength. As NaOH concentration increases, Na^+^ concentration also increases and, although these cations are highly hydrated, the increase in the ionic strength leads to a reduction of the double layer thickness at the particles’ surface, that affects their stability, inducing aggregation. In general, the large availability of monomers triggers the formation of larger MSNs, with a broader size distribution due both to monomer consumption and size-dependent aggregation. As a consequence, MSNs collected after 4 h ageing (MSN_H7, Figure 2A), show a smaller size and narrow size distribution, ascribed to a time-limited growth. A similar decrease in the average size can be observed by decreasing T_injection_. MSN_H8 (Figure 2B,E, Figure S3 in the Supplementary Materials), obtained in a synthesis performed at 30 °C, results in MSNs with average size of 73 nm and broad size distribution, that suggests the key role played by T_injection_ in the kinetic of MSN formation. 

As previously stated, the amount of H_2_O plays a pivotal role in the size regulation. TEM micrographs and size statistical analysis of sample MSN_H9 prepared under the same reaction conditions and with lower water volume (see Table 1), are reported in Figure 2C,F (Figure S3 in the Supplementary Materials), respectively, showing the formation of MSNs of nearly 102 nm (σ% = 17%), smaller than MSN_H6 (135 nm). It can be assumed that the higher H_2_O volume used for MSN_H6 results in an increase in the hydrolysis rate and higher amount of monomer supply and, therefore, larger MSNs are formed. Such an amount of H_2_O, does not act as a diluting solvent but, mainly, as reagent, thus affecting, with its concentration, the kinetic of the reaction. 

Irrespectively of the reaction medium, after several cycles of purification, MSN suspensions show a negative charge density and a ζ-potential value of −40.4 (±1.1) meV, that becomes slightly less negative, −34.2 (±0.6) meV, after template removal by alcoholic HCl solvent extraction. A more negative value, −56.9 (±1.6) meV has been, instead, measured after calcination. While calcination removes only the positive charged CTAB micelles, the solvent extraction under acidic condition contributes also helps to protonate, though partially, the outer exposed silanol groups, ultimately reducing the negative surface potential.

### 2.2. Synthesis of MSNs: A Two-Phase System Approach

When ethyl acetate replaces ethanol in the reaction mixture, a two-phase system forms, featuring ethyl acetate as upper phase with an aqueous bottom phase. TEOS is highly soluble in ethyl acetate and poorly dispersible in aqueous phase, therefore, the silica precursor can be assumed to hydrolyse at the interface between the two phases, and slowly be supplied to the aqueous bottom phase. The reaction scheme of such a synthetic approach is reported in Figure S4A.

**Figure 4:**
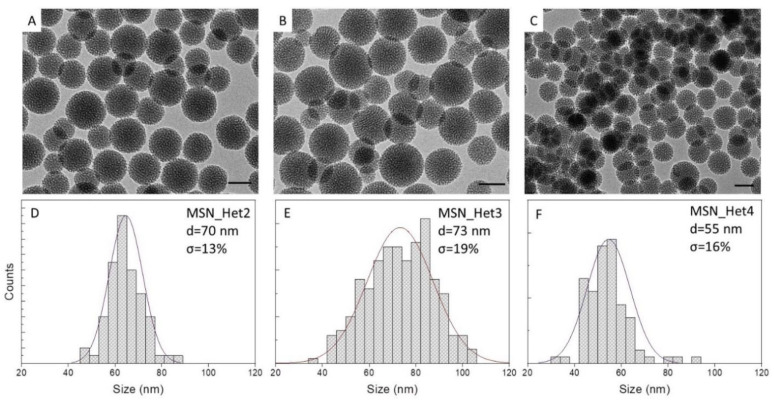
TEM micrographs (**A**–**C**, scale bar 50 nm) and the corresponding size distribution statistical analysis (**D**–**F**) of MSNs prepared by injecting TEOS 4.47 mmol (1 mL) at T_injection_ = 50 °C ((**A**,**B**,**D**,**E**) MSN_Het2 and MSN_Het3 samples) or T_injecttion_ = 30 °C ((**C**,**F**) MSN_Het4 sample) to 50 mL of H_2_O/Ethyl acetate (50:2 *v*/*v*), [CTAB] = 5 mM and [NaOH] 5 mM ((**A**,**D**) MSN_Het2) and 13 mM ((**B**,**C**,**E**,**F**), MSN_Het3, MSN_Het 4). t_reaction_ 3 h and t_ageing_ 24 h.

Table 2 reports a selection of samples prepared by using the two-phase approach, varying NaOH concentration in the range of 2 mM up to 13 mM, and keeping CTAB at a concentration of 5 mM, t_reaction_ of 3 h and t_ageing_ of 24 h. The experiments have been performed both at T_injection_ 50 °C and 30 °C (See Table 2). The TEM micrographs of the samples and their size statistical analysis are reported in Figure 4 and Figure S4B in the Supplementary Materials.

Spherical MSNs (Figure 4A, MSN_Het2), 70 nm in diameter (σ% = 13%) have been formed even using 5 mM NaOH. Such a NaOH concentration has been found insufficient to catalyse the particle synthesis exploiting the homogenous solution approach, that has required at least 8 mM NaOH. However, a further decrease in the NaOH concentration, down to 2 mM, prevents the formation of MSNs, in the two-phase system leading to the formation of an amorphous silica network (Figure S4B in the Supplementary Materials). 

As previously reported, CTAB aqueous solutions obtained using 5 mM and 13 mM NaOH exhibit a pH > 11, able to easily catalyse hydrolysis of TEOS and, in principle, sustain a large monomer supply. However, in the homogenous system, the addition of TEOS and ethanol and the fast hydrolysis result in a sudden consumption of hydroxyl ions and a pH decrease, for the 5 mM NaOH reaction mixture, that affect the kinetic of MSN synthesis and inhibiting the formation of MSNs. Conversely, in the two-phase system, the hydrolysis of TEOS slowly occurs at the interface between ethyl acetate and aqueous medium, and only slightly affects the pH of the aqueous bottom phase, thus a wide range of possible initial NaOH concentrations is suitable to achieve the convenient pH conditions for MSN synthesis. Such mechanism is also consistent with the evidence that the MSNs size remains almost unchanged, irrespectively from the initial NaOH concentration used (70 nm σ% = 13, for MSN_Het2 sample prepared at 5 mM NaOH and 74 nm σ% = 19, for MSN_Het3 sample prepared at 13mM NaOH), as highlighted by the TEM characterization (Figure 4A,B,D,E), thus suggesting that MSNs formation kinetic remain unchanged. FTIR characterization of these samples (Figure S4C in the Supplementary Materials) indicates a lower cross-linking degree for MSNs prepared using the two-phase system when compared to those prepared by means of the homogenous solution and a relative intensity of the Si-OH stretching vibration at 965 cm^−1^ higher than that of the MSN_H6 sample, thus suggesting the presence of a larger amount of silanol groups. Furthermore, significantly smaller size, is observed for MSN_Het3 (73 nm), prepared in the two-phase system, than MSN_H6 (135 nm), achieved in the homogenous solution, under the same reaction condition.

Such a result cannot be accounted for just by monomer accessibility, since, although the monomer release kinetics can be expected different in the two methods, in fact, the same amount of TEOS has been used, and is therefore, in principle, available, for the MSNs growth in both cases. However, since the mechanism supporting the MSN formation in the two-phase systems relies on nucleation and growth of particles at the interface, once they have reached certain size and weight, they leave the interface, where TEOS is supplied, to move down to the bulk of the aqueous bottom phase, where they stop growing. Therefore, according to such a mechanism, the initial NaOH concentration does not strictly determine the MSNs size. Conversely, temperature has been found to significantly affect the MSNs size. TEM micrographs of MSN_Het4, prepared at 30 °C, reported in Figure 4C (and statistical analysis in Figure 4F), highlights that a temperature decrease leads to MSNs smaller (average size 55 nm σ% = 16) than those attained at 50 °C. Temperature, through modification of kinetic energy, control the MSN sedimentation. Higher temperature can be assumed to make MSNs stay suspended longer at the interface, where monomer is supplied, and thus keep growing, while at low temperature they precipitate much earlier. 

### 2.3. MSNs Textural Properties

**Figure 5:**
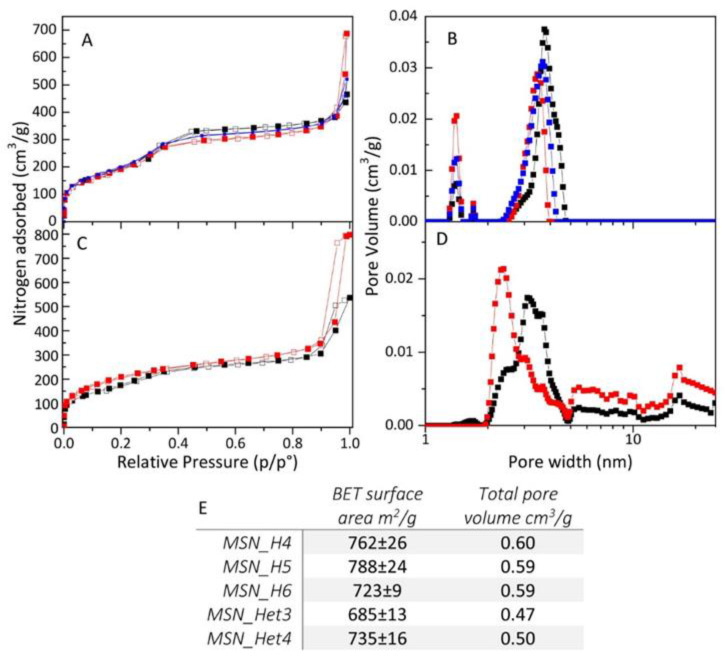
(**A**–**C**) N_2_ adsorption-desorption isotherms and (**B**,**D**) pore width distributions by NLDFT of MSN_H4 (panel A, B red line), MSN_H5 (panel A, B blue line), MSN_H6 (panel A, B black line), MSN_Het3 (panel C, D, black line), MSN_Het4 (panel C, D red line); (**E**) table of MSNs samples surface area and pore volume.

N_2_ adsorption and desorption isotherm and pore width distribution, as measured by non-local density functional theory (NLDFT) calculations, are reported in Figure 5 for samples prepared by the homogenous reaction solution as function of NaOH concentration (Figure 5A,B) and two-phase systems (Figure 5C,D) as function of reaction temperature. Structure and properties of the MSNs have been also evaluated by considering the role of MSN size and preparative conditions. 

All investigated samples are characterized by high specific surface area (SSA), showing Brunauer-Emmett-Teller (BET) surface area values in the range 680–780 m^2^/g. The MSNs prepared by homogeneous and two-phase approach mainly differ in their pore size distribution.

Nitrogen adsorption analysis results for MSNs obtained by the homogeneous solution approach, at different base concentration, are reported in Figure 5A,B. All samples show type IV isotherms [58] with a steep inflection between 0.3 and 0.4 p/p^0^, which is characteristic of mesoporous materials with a narrow pore size distribution (Figure 5A) [59]. The NLDFT model points out that porosity of these MSNs is characterized by a narrow range of pore width distribution (Figure 5B). In particular, increasing the base concentration from 8 mM to 13 mM, the isotherm inflection is shifted at higher relative pressures, indicating different ranges of porosity for the three samples. Indeed, MSN_H4, MSN_H5 and MSN_H6 present their main porosity in the ranges of 2.6–4, 2.5–4.3 and 3–4.5 nm, respectively. Also, increasing the base concentration, a decrease of the intensity of a minor porosity, centred at about 1.4 nm and attributed to semi-occluded pores, is recorded. 

Considering the samples from the two-phase approach, nitrogen adsorption for MSN_Het3 and MSN_Het4, obtained at T_injection_ 50 °C and 30 °C, respectively, show isotherms that significantly differ from those recorded for the samples prepared in homogenous solution and are also different from each other. MSN_Het3 presents a type IV isotherm characterized by a less sharp inflection with respect to the samples from the homogeneous solution synthesis, indicating a broader pore width distribution. Also, for this sample the isotherm inflection step is centred at lower relative pressure (about 0.2 p/p^0^), which indicates the presence of smaller mesopores. In fact, MSN_Het3 is characterized by pores with width ranges from 2 nm to 5 nm, with a major distribution peak centred at about 3 nm and a minor distribution peak centred at 2.5 nm. On the other hand, MSN_Het4 presents a nitrogen adsorption isotherm with significant adsorption at a pressure lower than that recorded for all the other MSNs isotherms, indicating the presence of narrower pores. Indeed, the NLDFT model shows for MSN_Het4 the presence of a major distribution peak centred at about 2.3 nm. The narrower pore size of MSN_Het4 consistently accounts for the higher BET surface area of this sample (735 m^2^/g) with respect to MSN_Het3 (685 m^2^/g), demonstrating that in the two-phase approach proposed, a decrease in the injection temperature from 50 °C to 30 °C induces the formation of MSNs characterized by narrower pores and higher specific surface area. For both the samples obtained from the two-phase approach, the pore width larger than 5 nm may be ascribed to interparticle capillary condensation, that also accounted for the isotherms’ hysteresis at high relative pressure. 

### 2.4. Assessment of MSNs Toxicity on Cell Viability of Endothelial Cell Line

The MSNs biocompatibility has been assessed considering their individual size and their surface chemistry, in terms of density of silanol groups. MSN_H4, MSN_H5, MSN_H6 and MSN_Het3 and MSN_Het4 have been selected and tested in the colorimetric (3-[4,5-dimethylthiazol-2-yl]-2,5 diphenyltetrazolium bromide) (MTT) assay, using an immortalized mouse brain-derived microvessel endothelial cell line, namely bEnd.3 cells. Previous studies have indeed indicated this cell line as a suitable model for investigating nanoparticle permeability [60] and furthermore may provide useful insights on possible biomedical application of the prepared MSNs, as a delivery system of diagnostic/therapeutic agents to the central nervous system. 

**Figure 6:**
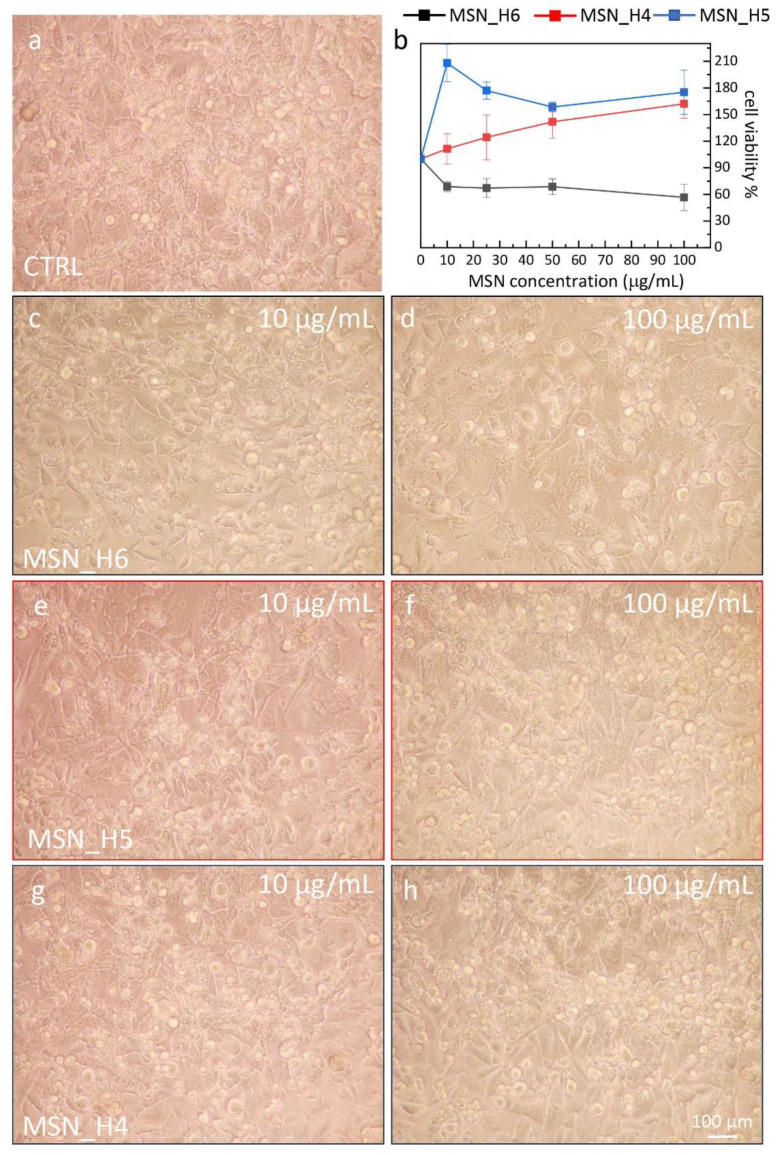
(**a**,**c**–**h**) Representative phase contrast optical images showing the morphology of bEnd.3 (20× magnification) and of the control (CTRL, 100%) after 24 h treatment with the MSN samples as labeled, prepared by the homogenous solution approaches, at the indicated concentrations. (**b**) Graph of the cell viability, determined by the MTT test, expressed as percentage of surviving cells in comparison with control (CTRL, 100%) represented by untreated bEnd.3 cells in serum-free DMEM.

**Figure 7:**
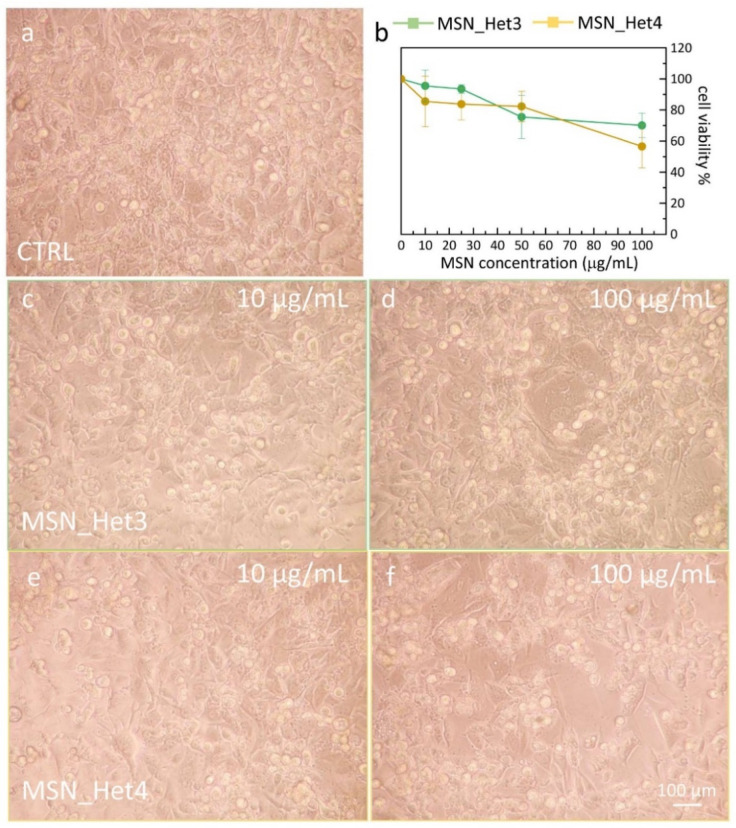
(**a**,**c**–**f**) Representative phase contrast optical images showing the morphology of bEnd.3 (20× magnification) and of the control (CTRL, 100%) after 24 h treatment with MSN samples as labeled, prepared by the two-phase approaches, at the indicated concentrations. (**b**) Graph of the cell viability, determined by the MTT test, expressed as percentage of surviving cells in comparison with control (CTRL, 100%) represented by untreated bEnd.3 cells in serum-free DMEM.

Cell viability has been evaluated by the MTT assay as function of MSN sample concentration, exploring a concentration range between 10–100 µg/mL. As shown in Figure 6b and Figure 7b, the cell viability of the bEnd.3 has been minimally affected upon their treatment for 24 h with MNS_H4 and MNS_H5. A slight dose-dependent decrease in the cell viability has been observed at the same extent for samples MSN_H6 and MSN_Het3 and MSN_Het4. This result can be accounted for by silanol groups, that in these samples are present in a high density, and can play a positive cytotoxic effect, inducing intracellular ROS formation responsible of cell oxidative damage. However, even at the highest tested concentration, 100 µg/mL, MSN_H6 and MSN_Het3 and MSN_Het4 cell viability above 50% has been always observed, indicating a poor cytotoxicity of the MSNs. 

The results do not show any remarkable in vitro toxic properties of the MSNs, and a response almost independent from the MSN size in this size regime. The morphology of bEnd.3 cells after their exposure to MNSs confirms the findings obtained by MTT assay; indeed, the endothelial cells show a round shape (rounding up), when incubated with the sample MSN_H6, MSN_Het3 and MSN_Het4 at the 100 µg/mL concentration, while retaining their elongated native morphology at low dose (10 µg/mL). Conversely, the morphological characterization of samples MSN_H5 and MSN_H4 reveals an increase in the cell density at higher MSN dose, highlighting that the MSNs do not significantly interfere with cell viability. 

## 3. Materials and Methods

### 3.1. Materials

Cetyl trimethylammonium bromide (CTAB > 96%), NaOH, tetraethyl orthosilicate (TEOS 98%) absolute ethanol (EtOH, 98%), ethyl acetate, HCl (32%) were purchased from Sigma-Aldrich (Milan, Italy). All aqueous solutions were prepared by using water obtained from a Milli-Q gradient A-10 system (Millipore, Bedford, MA, USA) 18.2 MΩcm, organic carbon content ≥4 µg/L). For cell viability test: Dulbecco’s Modified Eagle Medium (DMEM, Gibco, ThermoFisher Scientific, Waltham, MA, USA), phosphate buffer 10 mM (PBS), methylthiazolyldiphenyl-tetrazolium bromide (MTT).

### 3.2. Synthesis of MSNs: Homogenous Solution

MSNs were synthesized by a soft-template approach. Briefly, CTAB (5mM) was dissolved in an alkaline aqueous solution by NaOH using MilIipore water. NaOH at concentrations of 5, 8, 10 and 13 mM were tested to catalyze the sol-gel reaction. The solution was kept at 30 °C under stirring. Then, the temperature was raised up to 50 °C (T_injection_) and EtOH (2 mL) and TEOS (4.47 mmol, 1 mL) were added. The solution was let stirring at T_injection_ for (t_reaction_) 1 h or 3 h prior to be cooled down at room temperature and recovered after 4 hours, when it reached room temperature or let stirring for 24 h (t_ageing_). Table 1 summarizes the reaction conditions used for the performed experiments.

Repeated cycles of centrifugation at 15300 g at 4 °C for 20 min and redispersion in EtOH were carried out to remove unreacted precursors and surfactant. Solution extraction with alcoholic acidic solution based on HCl in EtOH at 0.156 M (V = 20 mL), was carried to remove the template. The solution was added to the MSN pellet, let sonicating for 3 hours, centrifuged and then the MSNs were recovered and redispersed in 5 mL of Millipore H_2_O. The final MSNs concentration was 38 mg/mL, as measured after freeze-drying. The as prepared samples were also thermally treated for 5 h at 500 °C.

### 3.3. Synthesis of MSNs: Two-Phase Approach

In the two phase-system, the MSNs were synthesized using CTAB (5 mM) as the template, dissolved in an alkaline aqueous solution (V = 50 mL), by NaOH, as a catalyst at different concentration (2 mM, 5 mM and 13 mM). TEOS was the silica source dissolved in ethyl acetate (2 mL). The solution was kept at 50 °C (T_injection_) under stirring and then, ethyl acetate (2 mL) and TEOS (4.47 mmol, 1 mL) were added. The solution was let to react for 3 h (t_reaction_) at the T_injection_ under vigorous stirring. The temperature was lowered to 25 °C and kept stirring for 24 h (t_ageing_). The reaction conditions are reported in Table 2. The as-prepared MSNs were purified with repeated cycles of centrifugation/redispersion in ethanol (13000 g at 18 °C for 1 h) to remove unreacted precursors and surfactant. The resulting solid product was dispersed in the alcoholic acidic solution (HCl in EtOH at 0.156 M, V = 20 mL) and let sonicated for 3 hours, centrifuged and then the MSNs were recovered, redispersed in 2 mL of Millipore H_2_O and freeze-dried, thus obtaining MSNs at a final concentration of 6 mg/mL. 

### 3.4. Characterization Techniques

Transmission electron microscopy (TEM) characterization was carried out using a JEM1011 instrument (JEOL, Akishima, Tokyo, Japan) operating at 100 keV, equipped with a high resolution CCD camera. Carbon-coated copper grids were dipped in the MSNs suspension 0.2mg/mL in EtOH and the solvent was let to evaporate. Statistical analysis of the features shown in the TEM micrographs were performed using the AxioVision image analysis freeware, and the average diameter of the MSNs was determined by measuring the diameter of nearly 150 and their size distribution calculated as percentage relative standard deviation (σ%) 

The FTIR characterization was carried out by using a 670 FTIR spectrometer (Varian, Palo Alto, CA, USA) equipped with a diamond ATR accessory of 2 mm and a deuterated tryglicine sulfate (DTGS) detector. One µL of each sample was put on the internal reflection element and the solvent was allowed to evaporate. Spectra were recorded in the range 4000–400 cm^−1^ acquiring 16 scans with a nominal resolution of 1 cm^−1^. 

For ζ- potential measurement, a Zetasizer nano ZSP (Malvern, USA) equipped with a laser diode that works at 50 mW and at a wavelength of 532 nm, was used. For the analysis, a solution of 7 μg/mL in filtered ultrapure water was used. 

Nitrogen adsorption analysis was performed on MSN to evaluate the textural properties of the nanocarriers. Brunauer-Emmett- Teller (BET) specific surface area was evaluated by N_2_ adsorption at 77 K through an ASAP 2020 analyzer (Micromeritics, Norcross, GA, USA). Pore size distribution was evaluated using the Non-local Density Functional Theory (NLDFT) analysis. The adsorption measurements were performed using high purity gases (>99.9.9%). MSN were degassed at 150 °C under vacuum before analysis (*p* < 10^−5^ mbar). 

### 3.5. MSN Cell Viability Test

For the cell viability tests MSNs were dispersed in PBS buffer and extensively sonicated. Brain derived endothelial cell lines bEnd.3, selected for MSN cell viability test, were seeded in 96 well plates, washed with serum-free DMEM and then treated with MSNs suspension at the concentrations of 10 μg/mL, 25 μg/mL, 50 μg/mL and 100 μg/mL. Control was represented untreated bEnd.3 in serum-free DMEM (CTRL). The treatment was performed in a final volume of 100 µL for 24 h at 37 °C, 5% CO_2_. At the end of the incubation period, cell culture supernatants were discarded and cells were subjected to the test of cell viability by MTT assay as reported [61]. Phase-contrast images of the cells treated with MSNs were also recorded and compared with the control.

## 4. Conclusions

Here, a systematic investigation on the tunability of MSN size in the sub-micrometer range using NaOH as base catalyst has been carried out by performing and comparing two synthetic approaches using a homogenous aqueous/ethanol solution and a two phase-system, obtained by replacing ethanol with ethyl acetate. All the reactions have been advantageously carried out at temperature not higher than 50 °C and using small reaction medium volumes. Such a simple reaction scheme, inspired by the Stöber approach, have been demonstrated to offer the potential of size tuning the MSNs in the sub-micrometer range by purposely balancing the reaction mixture composition and synthesis conditions. Different key factors have been demonstrated for each synthetic approach, able to regulate the MSNs size, thus suggesting that distinct mechanisms apply in the formation of the MSNs as a function of the reaction media. Morphological characterization has highlighted the pivotal role played by the NaOH concentration, reaction time and temperature and ageing time in the size tuning of MSNs prepared in homogenous solution, with a size control in the 40–150 nm range. Conversely, for the syntheses performed in the two-phase system, MSN size resulted mainly affected by the reaction temperature. Since particles nucleate and growth at the interface between aqueous phase and ethyl acetate, a higher temperature retards sedimentation. Smaller MSNs (55 nm) have been attained at 30 °C while larger MSNs (73 nm) at 50 °C. 

The porosity characterization highlighted for all the samples a high specific surface area > 680 m^2^/g. MSNs prepared in homogenous reaction media showed narrow pore width distributions with main porosity in the ranges of 2.6–4.0, 2.5–4.3 and 3–4.5 nm, respectively at increasing base concentration.

Conversely, MSNs synthesized in a two-phase system were characterized by a broader pore width distribution, mainly in the range 2–5 nm. In this case, the MSNs’ pore width was found dependent on the reaction temperature, as it decreased with the temperature decreasing from 50 to 30 °C. 

The in vitro study performed on the endothelial bEnd.3 cells has revealed a high degree of biocompatibility of the different prepared MNSs. A slight decrease of the cell viability has been observed only for those samples of MSNs showing the highest density of silanol groups. In fact, in this case, upon incubation, cell damage induced by oxidative stress due to ROS generation can reasonably take place to a larger extent.

The presented systematic morphological and structural characterization and the in-depth investigation of the MSN synthetic mechanism catalysed by NaOH, provide practical guidelines for an effective and fine MSN size control in the sub-micrometre regime, that is crucial for biomedical applications, but remains not straightforward when such a base catalyst is exploited, due to the fast hydrolysis and uncontrolled aggregation phenomena.

## Figures and Tables

**Table 1:** Reaction conditions used for the MSNs synthesis following the homogenous solution approach and average size and size distribution of the obtained samples, determined by TEM characterization. (*) and (**) correspond to amorphous structures and barely defined spherical particles, respectively (see Figure S1 in the Supplementary Materials).

	T_injection_ °C	t_reaction_ h	t_ageing_ h	[NaOH] mM	H_2_O/EtOH Volume Ratio	Average Size nm	Size Distribution σ%
*MSN_H1*	50	3	24	5	150:2	*	---
*MSN_H2*	50	3	24	5	50:2	**	---
*MSN_H3*	50	1	24	8	50:2	35	11
*MSN_H4*	50	3	24	8	50:2	47	13
*MSN_H5*	50	3	24	10	50:2	64	15
*MSN_H6*	50	3	24	13	50:2	135	17
*MSN_H7*	50	3	4	13	50:2	54	11
*MSN_H8*	30	3	24	13	50:2	73	16
*MSN_H9*	50	3	24	13	30:2	102	16

**Table 2:** Reaction conditions for the MSNs synthesized in a two-phase system and average size and size distribution of the resulting particles determined from the TEM analysis. (*) As shown in Figure S4B in the Supplementary Materials a siliceous network rather than MSNs forms.

	T_injection_ °C	t_reaction_ Hours	t_ageing_ Hours	[NaOH] mM	H_2_O/Ethyl Acetate Volume Ratio	Average Size nm	Size Distribution σ%
*MSN_Het1*	50	3	24	2	50:2	*	---
*MSN_Het2*	50	3	24	5	50:2	70	13
*MSN_Het3*	50	3	24	13	50:2	73	19
*MSN_Het4*	30	3	24	13	50:2	55	16

## Data Availability

The data presented in this study are available in Supplementary Material.

## Supplementary Materials

The following are available online. Figure S1 reports the TEM micrographs of MSN samples prepared at 5 mM NaOH concentration in a homogenous aqueous/ethanol solution in the presence of CTAB micelles, as structure-directing agent, and TEOS, as silica source; Figure S2 reports the size statistical analysis of samples in Figure 1B-E in the main manuscript; Figure S3 shows the TEM microphase of MSN_H5, MSN_H6, MSN_H8, MSN_H9; Figure S4 shows the reaction scheme for the two-phase system synthesis of MSNs, TEM micrograph at 2 mM NaOH concentration and the FT-IR in ATR mode of the MSN_Het2 and MSN_Het3 samples.

## Sample Availability

Samples of the compounds are not available from the authors.

## References

[1] Jarmolińska S., Feliczak-Guzik A., Nowak I. (2020). Synthesis, Characterization and Use of Mesoporous Silicas of the Following Types SBA-1, SBA-2, HMM-1 and HMM-2. Materials.

[2] Olivieri F., Castaldo R., Cocca M., Gentile G., Lavorgna M. (2021). Mesoporous silica nanoparticles as carriers of active agents for smart anticorrosive organic coatings: A critical review. Nanoscale.

[3] Pal N., Lee J.-H., Cho E.-B. (2020). Recent Trends in Morphology-Controlled Synthesis and Application of Mesoporous Silica Nanoparticles. Nanomaterials.

[4] von Baeckmann C., Guillet-Nicolas R., Renfer D., Kählig H., Kleitz F. (2018). A Toolbox for the Synthesis of Multifunctionalized Mesoporous Silica Nanoparticles for Biomedical Applications. ACS Omega.

[5] Jafari S., Derakhshankhah H., Alaei L., Fattahi A., Varnamkhasti B.S., Saboury A.A. (2019). Mesoporous silica nanoparticles for therapeutic/diagnostic applications. Biomed. Pharmacother..

[6] Niculescu V.-C. (2020). Mesoporous Silica Nanoparticles for Bio-Applications. Front. Mater..

[7] Da’na E. (2017). Adsorption of heavy metals on functionalized-mesoporous silica: A review. Microporous Mesoporous Mater..

[8] Singh B., Na J., Konarova M., Wakihara T., Yamauchi Y., Salomon C., Gawande M.B. (2020). Functional Mesoporous Silica Nanomaterials for Catalysis and Environmental Applications. Bull. Chem. Soc. Jpn..

[9] Sheng W., Wei W., Li J., Qi X., Zuo G., Chen Q., Pan X., Dong W. (2016). Amine-functionalized magnetic mesoporous silica nanoparticles for DNA separation. Appl. Surf. Sci..

[10] Motahar S., Nikkam N., Alemrajabi A.A., Khodabandeh R., Toprak M.S., Muhammed M. (2014). A novel phase change material containing mesoporous silica nanoparticles for thermal storage: A study on thermal conductivity and viscosity. Int. Commun. Heat Mass Transf..

[11] Castaldo R., de Luna M.S., Siviello C., Gentile G., Lavorgna M., Amendola E., Cocca M. (2020). On the acid-responsive release of benzotriazole from engineered mesoporous silica nanoparticles for corrosion protection of metal surfaces. J. Cult. Herit..

[12] Florek J., Caillard R., Kleitz F. (2017). Evaluation of mesoporous silica nanoparticles for oral drug delivery—Current status and perspective of MSNs drug carriers. Nanoscale.

[13] Stöber W., Fink A., Bohn E. (1968). Controlled growth of monodisperse silica spheres in the micron size range. J. Colloid Interface Sci..

[14] Tsai C.-H., Vivero-Escoto J.L., Slowing I.I., Fang I.J., Trewyn B.G., Lin V.S.Y. (2011). Surfactant-assisted controlled release of hydrophobic drugs using anionic surfactant templated mesoporous silica nanoparticles. Biomaterials.

[15] Zhang K., Xu L.-L., Jiang J.-G., Calin N., Lam K.-F., Zhang S.-J., Wu H.-H., Wu G.-D., Albela B., Bonneviot L. (2013). Facile Large-Scale Synthesis of Monodisperse Mesoporous Silica Nanospheres with Tunable Pore Structure. J. Am. Chem. Soc..

[16] Han L., Gao C., Wu X., Chen Q., Shu P., Ding Z., Che S. (2011). Anionic surfactants templating route for synthesizing silica hollow spheres with different shell porosity. Solid State Sci..

[17] Kruk M. (2012). Access to Ultralarge-Pore Ordered Mesoporous Materials through Selection of Surfactant/Swelling-Agent Micellar Templates. Acc. Chem. Res..

[18] Liu J., Hartono S.B., Jin Y.G., Li Z., Lu G.Q., Qiao S.Z. (2010). A facile vesicle template route to multi-shelled mesoporous silica hollow nanospheres. J. Mater. Chem..

[19] Li N., Niu D., Jiang Y., Xu C., Pan S., He J., Chen J., Zhang L., Li Y. (2017). Morphology Evolution and Spatially Selective Functionalization of Hierarchically Porous Silica Nanospheres for Improved Multidrug Delivery. Chem. Mater..

[20] Eltohamy M., Shin U.S., Kim H.-W. (2011). Silica nanoparticles with enlarged nanopore size for the loading and release of biological proteins. Mater. Lett..

[21] Huang L., Kruk M. (2015). Versatile Surfactant/Swelling-Agent Template for Synthesis of Large-Pore Ordered Mesoporous Silicas and Related Hollow Nanoparticles. Chem. Mater..

[22] Niu D., Liu Z., Li Y., Luo X., Zhang J., Gong J., Shi J. (2014). Monodispersed and Ordered Large-Pore Mesoporous Silica Nanospheres with Tunable Pore Structure for Magnetic Functionalization and Gene Delivery. Adv. Mater..

[23] Wei J., Sun Z., Luo W., Li Y., Elzatahry A.A., Al-Enizi A.M., Deng Y., Zhao D. (2017). New Insight into the Synthesis of Large-Pore Ordered Mesoporous Materials. J. Am. Chem. Soc..

[24] Han C., Huang H., Dong Y., Sui X., Jian B., Zhu W. (2019). A Comparative Study of the Use of Mesoporous Carbon and Mesoporous Silica as Drug Carriers for Oral Delivery of the Water-Insoluble Drug Carvedilol. Molecules.

[25] Moodley T., Singh M. (2020). Sterically Stabilised Polymeric Mesoporous Silica Nanoparticles Improve Doxorubicin Efficiency: Tailored Cancer Therapy. Molecules.

[26] Slowing I.I., Wu C.-W., Vivero-Escoto J.L., Lin V.S.-Y. (2009). Mesoporous Silica Nanoparticles for Reducing Hemolytic Activity Towards Mammalian Red Blood Cells. Small.

[27] Mozafarinia M., Karimi S., Farrokhnia M., Esfandiari J. (2021). In vitro breast cancer targeting using Trastuzumab-conjugated mesoporous silica nanoparticles: Towards the new strategy for decreasing size and high drug loading capacity for drug delivery purposes in MSN synthesis. Microporous Mesoporous Mater..

[28] Chen S., Hu J., Wang F., Liu H. (2019). Preparation and drug release application of pH and light dual-stimuli- responsive nanocarrier based on mesoporous silica nanoparticles. J. Dispers. Sci. Technol..

[29] Jadhav S.A., Miletto I., Brunella V., Berlier G., Scalarone D. (2015). Controlled post-synthesis grafting of thermoresponsive poly(*N*-isopropylacrylamide) on mesoporous silica nanoparticles. Polym. Adv. Technol..

[30] Ferris D.P., Lu J., Gothard C., Yanes R., Thomas C.R., Olsen J.-C., Stoddart J.F., Tamanoi F., Zink J.I. (2011). Synthesis of Biomolecule-Modified Mesoporous Silica Nanoparticles for Targeted Hydrophobic Drug Delivery to Cancer Cells. Small.

[31] Hu X., Wang Y., Peng B. (2014). Chitosan-Capped Mesoporous Silica Nanoparticles as pH-Responsive Nanocarriers for Controlled Drug Release. Chem. An. Asian J..

[32] Mondragón L., Mas N., Ferragud V., de la Torre C., Agostini A., Martínez-Máñez R., Sancenón F., Amorós P., Pérez-Payá E., Orzáez M. (2014). Enzyme-responsive intracellular-controlled release using silica mesoporous nanoparticles capped with ε-poly-L-lysine. Chemistry.

[33] Xu P., Guo S., Yu H., Li X. (2014). Mesoporous Silica Nanoparticles (MSNs) for Detoxification of Hazardous Organophorous Chemicals. Small.

[34] Luo Z., Hu Y., Xin R., Zhang B., Li J., Ding X., Hou Y., Yang L., Cai K. (2014). Surface functionalized mesoporous silica nanoparticles with natural proteins for reduced immunotoxicity. J. Biomed. Mater. Res. Part A.

[35] Ribes À., Aznar E., Bernardos A., Marcos M.D., Amorós P., Martínez-Máñez R., Sancenón F. (2017). Fluorogenic Sensing of Carcinogenic Bisphenol A using Aptamer-Capped Mesoporous Silica Nanoparticles. Chemistry.

[36] Palanikumar L., Choi E.S., Cheon J.Y., Joo S.H., Ryu J.-H. (2015). Noncovalent Polymer-Gatekeeper in Mesoporous Silica Nanoparticles as a Targeted Drug Delivery Platform. Adv. Funct. Mater..

[37] Cao L., Zhang H., Cao C., Zhang J., Li F., Huang Q. (2016). Quaternized Chitosan-Capped Mesoporous Silica Nanoparticles as Nanocarriers for Controlled Pesticide Release. Nanomaterials.

[38] Sun T., Sun Y., Zhang H. (2018). Phospholipid-Coated Mesoporous Silica Nanoparticles Acting as Lubricating Drug Nanocarriers. Polymers.

[39] Melendez-Rodriguez B., Figueroa-Lopez K.J., Bernardos A., Martínez-Máñez R., Cabedo L., Torres-Giner S., Lagaron J.M. (2019). Electrospun Antimicrobial Films of Poly(3-hydroxybutyrate-co-3-hydroxyvalerate) Containing Eugenol Essential Oil Encapsulated in Mesoporous Silica Nanoparticles. Nanomaterials.

[40] Meka A.K., Jenkins L.J., Dàvalos-Salas M., Pujara N., Wong K.Y., Kumeria T., Mariadason J.M., Popat A. (2018). Enhanced Solubility, Permeability and Anticancer Activity of Vorinostat Using Tailored Mesoporous Silica Nanoparticles. Pharmaceutics.

[41] Shan Y., Cao L., Xu C., Zhao P., Cao C., Li F., Xu B., Huang Q. (2019). Sulfonate-Functionalized Mesoporous Silica Nanoparticles as Carriers for Controlled Herbicide Diquat Dibromide Release through Electrostatic Interaction. Int. J. Mol. Sci..

[42] Kim M.-K., Ki D.-H., Na Y.-G., Lee H.-S., Baek J.-S., Lee J.-Y., Lee H.-K., Cho C.-W. (2021). Optimization of Mesoporous Silica Nanoparticles through Statistical Design of Experiment and the Application for the Anticancer Drug. Pharmaceutics.

[43] Kumari S., Sahare P.D. (2014). Photoluminescence studies of stilbene laser dye incorporated mesoporous silica nanoparticle (MSN) with sulphur dioxide. J. Porous Mater..

[44] Xi C., Zhou J., Du S., Peng S. (2016). Autophagy upregulation promotes macrophages to escape mesoporous silica nanoparticle (MSN)-induced NF-κB-dependent inflammation. Inflamm. Res..

[45] Garrido-Cano I., Candela-Noguera V., Herrera G., Cejalvo J.M., Lluch A., Marcos M.D., Sancenon F., Eroles P., Martínez-Máñez R. (2021). Biocompatibility and internalization assessment of bare and functionalised mesoporous silica nanoparticles. Microporous Mesoporous Mater..

[46] Díaz-García D., Sommerova L., Martisova A., Skoupilova H., Prashar S., Vaculovic T., Kanicky V., del Hierro I., Hrstka R., Gómez-Ruiz S. (2020). Mesoporous silica nanoparticles functionalized with a dialkoxide diorganotin(IV) compound: In search of more selective systems against cancer cells. Microporous Mesoporous Mater..

[47] Katagiri K., Yamazaki S.-I., Inumaru K., Koumoto K. (2015). Anti-reflective coatings prepared via layer-by-layer assembly of mesoporous silica nanoparticles and polyelectrolytes. Polym. J..

[48] Luo G.-F., Chen W.-H., Liu Y., Lei Q., Zhuo R.-X., Zhang X.-Z. (2014). Multifunctional Enveloped Mesoporous Silica Nanoparticles for Subcellular Co-delivery of Drug and Therapeutic Peptide. Sci. Rep..

[49] Zeng D., Zhang H., Wang B., Sang K., Yang J. (2015). Effect of Ammonia Concentration on Silica Spheres Morphology and Solution Hydroxyl Concentration in Stober Process. J. Nanosci. Nanotechnol..

[50] Chen Y., Chen H., Guo L., He Q., Chen F., Zhou J., Feng J., Shi J. (2010). Hollow/Rattle-Type Mesoporous Nanostructures by a Structural Difference-Based Selective Etching Strategy. ACS Nano.

[51] Chen Q., Wu W.B., Mak C.L., Wong K.H. (2001). Growth of highly oriented of Pb(Zrx, Ti_1−x_)O_3_ film on porous silicon. Thin Solid Film..

[52] Warring S.L., Beattie D.A., McQuillan A.J. (2016). Surficial Siloxane-to-Silanol Interconversion during Room-Temperature Hydration/Dehydration of Amorphous Silica Films Observed by ATR-IR and TIR-Raman Spectroscopy. Langmuir.

[53] Kaya H., Ngo D., Gin S., Kim S.H. (2020). Spectral changes in Si–O–Si stretching band of porous glass network upon ingress of water. J. Non-Cryst. Solids.

[54] Almeida R.M., Marques A.C. (2016). Characterization of Sol–Gel Materials by Infrared Spectroscopy. Handbook of Sol-Gel Science and Technology.

[55] Capeletti L.B., Zimnoch J.H. (2016). Fourier Transform Infrared and Raman Characterization of Silica-Based Materials. Applications of Molecular Spectroscopy to Current Research in the Chemical and Biological Sciences.

[56] Wu S.-H., Mou C.-Y., Lin H.-P. (2013). Synthesis of mesoporous silica nanoparticles. Chem. Soc. Rev..

[57] Ghimire P.P., Jaroniec M. (2021). Renaissance of Stöber method for synthesis of colloidal particles: New developments and opportunities. J. Colloid Interface Sci..

[58] Thommes M., Kaneko K., Neimark A.V., Olivier J.P., Rodriguez-Reinoso F., Rouquerol J., Sing K.S.W. (2015). Physisorption of gases, with special reference to the evaluation of surface area and pore size distribution (IUPAC Technical Report). Pure Appl. Chem..

[59] Grun M., Matsumoto K.K.U.A., Tsutsumi K. (1999). Novel Pathways for the Preparation of Mesoporous Mcm-41 Materials—Control of Porosity and Morphology. Micropor. Mesopor. Mater..

[60] Norouzi M., Yathindranath V., Thliveris J.A., Kopec B.M., Siahaan T.J., Miller D.W. (2020). Doxorubicin-loaded iron oxide nanoparticles for glioblastoma therapy: A combinational approach for enhanced delivery of nanoparticles. Sci. Rep..

[61] Di Bari G., Gentile E., Latronico T., Corriero G., Fasano A., Nonnis Marzano C., Liuzzi G.M. (2015). Inhibitory Effect of Aqueous Extracts from Marine Sponges on the Activity and Expression of Gelatinases A (MMP-2) and B (MMP-9) in Rat Astrocyte Cultures. PLoS ONE.

