# Supplementary material for: High Surface Area Mesoporous Silica Nanoparticles with Tunable Size in the Sub-Micrometer Regime: Insights on the Size and Porosity Control Mechanisms
Source: Molecules. 2021 Jul 13;26(14):4247. doi: 10.3390/molecules26144247 (PMC8304748; doi:10.3390/molecules26144247)
Supplement: Supplementary file 1 [file molecules-26-04247-s001.zip › molecules-1286734-supplementary.pdf]

molecules

MDPI

Supplementary Material

High Surface Area Mesoporous Silica Nanoparticles with Tunable Size in the Sub-Micrometer Regime: Insights on the Size and Porosity Control Mechanisms

Federica Rizzi [1,2], Rachele Castaldo [3], Tiziana Latronico [4], Pierluigi Lasala [1], Gennaro Gentile [3], Marino Lavorgna [5], Marinella Striccoli [2], Angela Agostiano [1,2], Roberto Comparelli [2], Nicoletta Depalo [2], Maria Lucia Curri [1,2,*] and Elisabetta Fanizza [1,2,*]

[1] Department of Chemistry, University of Bari, Via Orabona 4, 70126 Bari, Italy; federica.rizzi@uniba.it (F.R.); pierluigi.lasala95@gmail.com (P.L.); angela.agostiano@uniba.it (A.A.)
[2] Institute for Physical Processes, Italian National Research Council, c/o Department of Chemistry, University of Bari, Via Orabona 4, 70126 Bari, Italy; m.striccoli@ba.ipcf.cnr.it (M.S.); r.comparelli@ba.ipcf.cnr.it (R.C.); n.depalo@ba.ipcf.cnr.it (N.D.)
[3] Institute for Polymers, Composites and Biomaterials, Italian National Research Council, Via Campi Flegrei 34, Pozzuoli, 80078 Naples, Italy; rachele.castaldo@ipcb.cnr.it (R.C.); gennaro.gentile@ipcb.cnr.it (G.G.)
[4] Department of Bioscience, Biotechnology and Biopharmaceutics, University of Bari, Via Orabona 4, 70126 Bari, Italy; tiziana.latronico@uniba.it
[5] Institute for Polymers, Composites and Biomaterials, Italian National Research Council, Piazzale E. Fermi 1, Portici, 80055 Naples, Italy; marino.lavorgna@cnr.it
* Correspondence: marialucia.curri@uniba.it (M.L.C.); elisabetta.fanizza@uniba.it (E.F.)

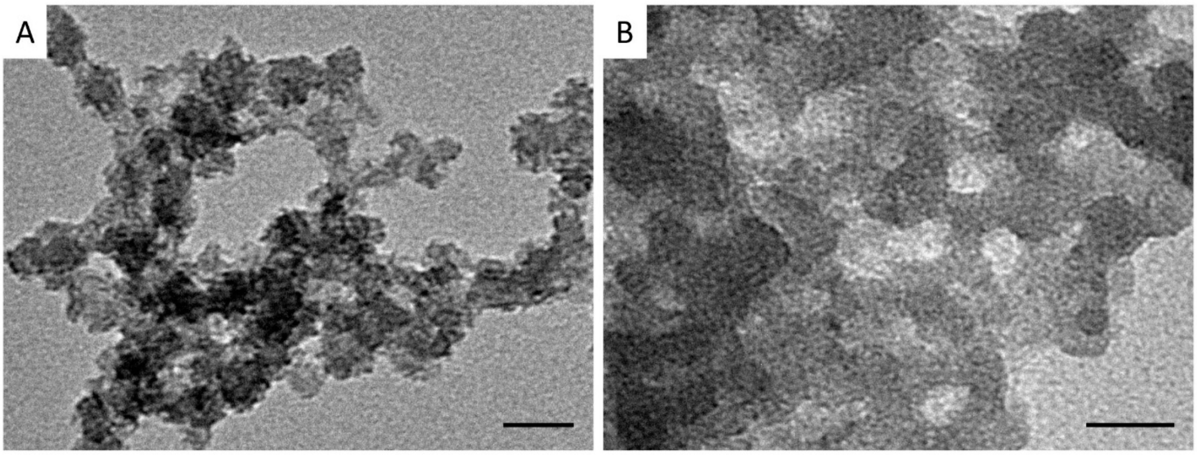

Figure S1. TEM micrographs (scale bar 25 nm) of MSNs prepared by injecting TEOS 4.47 mmol (1 mL) at $T_{injection}$= 50°C to 150 mL of H_2O/EtOH (50:2 v/v) (A, sample MSN_H2) and to 50 mL of H_2O/EtOH (150:2 v/v) (B, sample MSN_H1), [CTAB]= 5 mM, $t_{reaction}$ =3 hours and t_{ageing} = 24 hours.

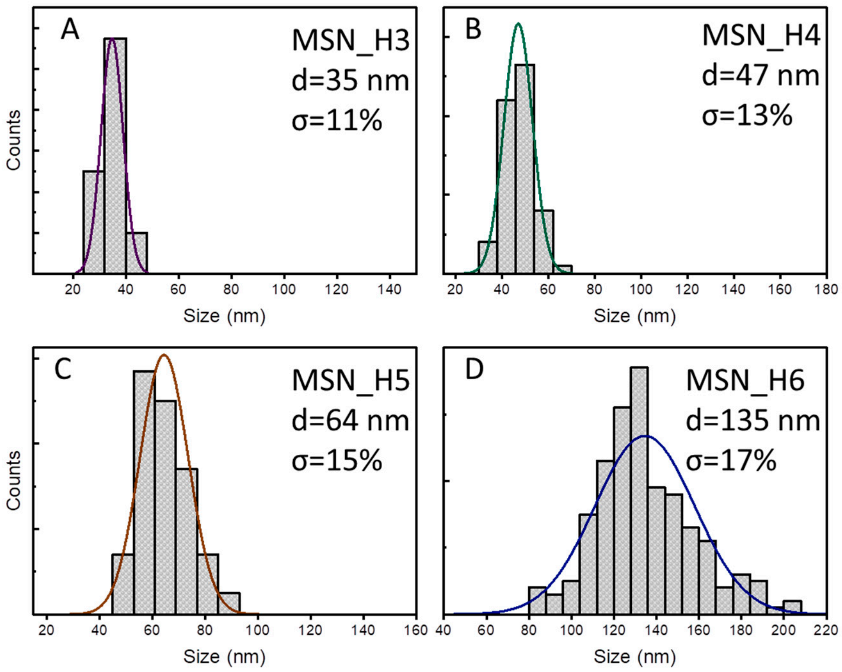

Figure S2. Size distribution statistical analysis (A-D, samples MSN_H3, MSN_H4, MSN_H5 and MSN_H6 samples) of MSNs prepared by injecting TEOS 4.47 mmol (1 mL) at $T_{injection}$= 50°C to 50 mL of H_2O/EtOH (50:2 v/v), [CTAB]=5 mM and [NaOH] 8 mM (A, B), 10 mM (C), 13 mM (D). $t_{reaction}$ kept at 1hour (B) and 3hours (B-D) and t_{ageing} of 24 hours.

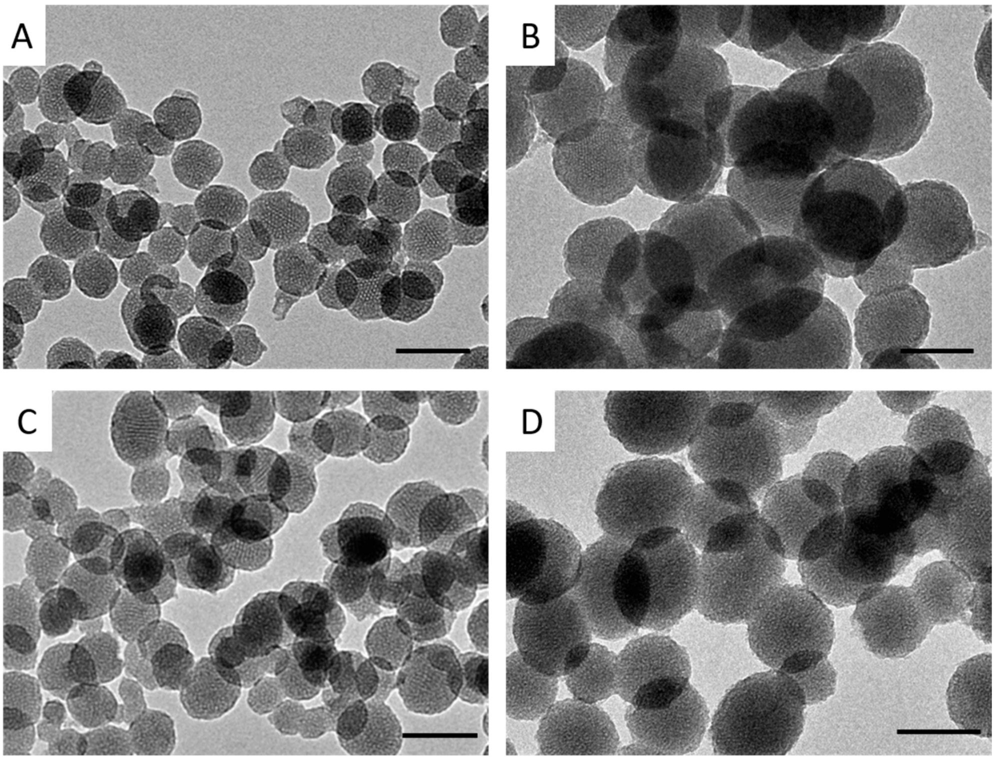

Figure S3. TEM micrographs (scale bar 100 nm) of MSNs prepared by injecting TEOS 4.47 mmol (1 mL) at $T_{injection}$= 50°C (A, B and D, samples MSN_H5, MSN_H6 and MSN_H9) and 30°C (C, sample MSN_H8) to 50 mL of H_2O/EtOH (50:2 v/v, A, B and C) or H_2O/EtOH (30:2 v/v, D), [CTAB]=5 Mm and [NaOH] = 10 mM (A), 13 mM (B, C and D), $t_{reaction}$ =3 hours and t_{ageing} at 24 hours

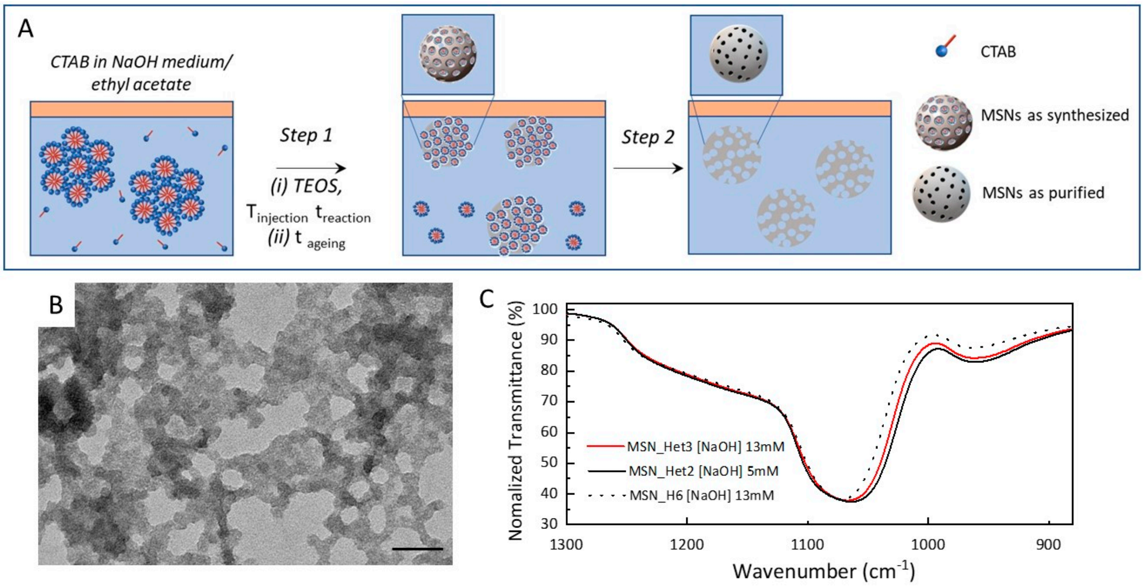

Figure S4. (A) Sketch of the two-phase synthetic approach of mesoporous silica nanoparticles (MSNs): (step 1) i. Tetraethyl orthosilicate (TEOS) addition at temperature $T_{injection}$ to H_2O/Ethyl acetate reaction mixture at alkaline pH by NaOH in the presence of CTAB micelles. Reaction is let to proceed for 3hours ($t_{reaction}$) then ii) reaction mixture is stirred at room temperature for 24 hours (t_{ageing}), (step 2) surfactant template removal by solvent extraction; (B) TEM micrograph (scale bar 50 nm) of synthesis carried out by injecting TEOS 4.47 mmol (1 mL) at $T_{injection}$= 50°C to 50 mL of H_2O/Ethyl acetate (50:2 v/v), [CTAB]=5 mM and [NaOH] 2 mM; (C) FTIR spectra in ATR mode in the 1300-880 cm^{-1} range of MSNs samples prepared in the two-phase system at different NaOH concentration (red and black solid line) compared to the MSNs sample prepared in homogenous solution (dashed line). The samples prepared by the two-phase systems show a a more open structure with lower compressive stress, and a lower cross-linking degree compared to MSN_H6 sample.
